# Dimerization Constants from Acoustic Measurements: Solutions of Benzene, Cyclohexylamine and Aniline in Cyclohexane

**DOI:** 10.1007/s10953-017-0656-1

**Published:** 2017-07-18

**Authors:** Andrzej Burakowski, Jacek Gliński

**Affiliations:** Faculty of Chemistry, University of Wrocław, F. Joliot-Curie 14, 50-383 Wrocław, Poland

**Keywords:** Sound speed, Density, Compressibility, Liquid mixtures, Association, Dimerization, Chemical equilibrium

## Abstract

A model assuming that the formation of dimers determines the acoustic properties of liquid mixtures, in the inert solvent cyclohexane, was applied to describe the observed dependences of sound speed on composition. The dimerization constants were estimated. The results allow one to propose that the solutes tend to form associates larger than dimers in concentrated solutions, while in dilute systems solute–solvent interactions play an important role.

**Electronic supplementary material:**

The online version of this article (doi:10.1007/s10953-017-0656-1) contains supplementary material, which is available to authorized users.

## Introduction

Sound speeds in liquids combined with their densities are often used to calculate the adiabatic compressibility coefficients, by applying the Laplace equation:

1 $$ \kappa_{S} = \frac{1}{{\rho \cdot c^{2} }} $$

where $\kappa_{S} = - \frac{1}{V}\left( {\frac{\partial V}{\partial p}} \right)_{S}$ is the isentropic (adiabatic) compressibility coefficient, *c* the sound speed and *ρ* the density, while subscript *S* denotes isentropic (adiabatic) condition. The compressibility of a liquid system is strongly dependent on the intermolecular interactions as well as the microscopic structure of the medium. However, with only a few exceptions, attempts to apply acoustic parameters, including compressibility, to investigate intermolecular interactions in, and the structure of, liquid systems are rare and usually limited to analyzing the deviations of experimental values from additivity or ideality [1].

Recently, we presented a simple model [2], with only two adjustable parameters, which treats a polar solute dissolved in an inert solvent as a mixture of its monomeric and dimeric forms only, while neglecting both solute–solvent interactions and the formation of higher associates. This model was successfully tested using data for alcohol–hydrocarbon solutions.

In this paper we present results for the title systems, interpreted in terms of the above model. The dimerization constants of solutes in cyclohexane solutions and their temperature dependences were determined, together with resulting thermodynamic quantities. Moreover, conclusions were drawn concerning other association processes occurring in the systems under investigation.

## The Model

As mentioned above, this model exploits the idea described in [2]. Let us assume that in a binary liquid solution, containing an inert, non-polar solvent and a polar solute, the only process other than solvation occurring is the equilibrium dimerization reaction, 2*A* ⇌ *A*_2_. This equilibrium can be described by the dimerization constant:

2 $$ K_{\dim } = \frac{{[A_{2} ]}}{{[A]^{2} }} $$

The brackets denote activities, which can be replaced by molar concentrations or mole fractions. We will use the latter concentration units here. Obviously the model, which is limited to dimerization as the only process occurring in polar component + non-polar hydrocarbon mixtures, is very rough and involves sometimes disputable simplifications. However, more realistic models, including formation of associates bigger than dimers or competitive solute–solvent associates of different sizes, would require many additional fitted parameters, causing their physical interpretation to be questionable.

According to the model, a mixture of polar (*A*) and non-polar (*B*) liquids contains only three components: the non-associated form of *A*, its associated, dimeric form *A*_2_, and the inert solvent *B*. Neither the mutual compositions of *A* and *A*_2_ nor the speed of sound of these pure forms are initially known.

It is well known that associated liquids are characterized by higher sound speeds than non-associated ones [3]. This allows one to assume that the sound velocity of the pure hypothetical monomeric form *A* is lower than in its dimeric form *A*_2_. The mutual compositions of these two forms can be easily determined from the dimerization constant *K*_dim_ (if known).

Here we employ the idea of Natta-Baccaredda, who assumed the additivity of sound propagation times in solutions with volume fractions of the components [4]. This idea was further tested and developed in [5]. It was found that it describes surprisingly well the sound speeds in liquid mixtures in which non-specific interactions prevail. According to this model, the observed sound speed in a liquid mixture is given by:

3 $$ c_{\text{calc}} = \frac{{c_{1} \cdot c_{2} \cdot c_{3} \ldots c_{n} }}{{\phi_{1} \cdot c_{2} \cdot c_{3} \ldots c_{n} + \phi_{2} \cdot c_{1} \cdot c_{3} \cdot c_{4} \ldots c_{n} + \ldots + \phi_{n} \cdot c_{1} \cdot c_{2} \cdot c_{3} \ldots c_{n - 1} }} $$

where *c*_calc_ is the calculated speed of sound in a mixture of *n* liquids, *ϕ*_*i*_ the volume fraction of pure *i*th component, and *c*_*i*_ the sound speed in the pure *i*th component.

Now, for *n* = 3 (hypothetical monomeric form *A*, hypothetical dimeric form *A*_2_, and inert solvent *B*) the experimental data set of sound velocities and compositions can be described with only two unknown parameters: *K*_dim_ and the speed of sound in one of the hypothetical forms of the polar solute, *A* or *A*_2_. Instead of the latter, the difference of the sound velocities in pure *A*_2_ and *A*, ∆*c* = $c_{\text{A}_2}$ *c*_A_ can be used. Knowing ∆*c* and *K*_dim_, one can calculate the values of $c_{\text{A}_2}$ and *c*_A_ from experimental sound speeds in the pure polar components of the mixture. Now, the relation of Natta–Baccaredda has the form:

4 $$ c_{\text{calc}} = \frac{{c_{A} \cdot c_{{A_{2} }} \cdot c_{B} }}{{\phi_{A} \cdot c_{{A_{2} }} \cdot c_{B} + \phi_{{A_{2} }} \cdot c_{A} \cdot c_{B} + \phi_{B} \cdot c_{A} \cdot c_{{A_{2} }} }} $$

A commercial computer program was sufficient to fit *K*_dim_ and, further, the volume fractions of the hypothetical phases with ∆*c* changed until a satisfactory fitting was reached, using Eq. 4 and speed of sound data versus concentration data, and the sum of squares of deviations ∆*c* = *c*_*i*,exp−_ *c*_*i*,calc_ is minimized during this fitting. The details of the algorithm are precisely, point-by-point, described in a paper already cited [2].

This procedure is similar to those applied for aqueous systems, where the compressibility (calculated using Eq. 1) or the acoustic impedance were assumed to be additive with the mole fractions of components and the solvated solute was treated as the third, non-compressible, component [6–9].

## Experimental

Cyclohexane (Sigma–Aldrich, pure, >99.5%), benzene (Lach-Ner, 99.94%), aniline (Alfa Aesar, 99+ %) and cyclohexylamine (Merck, >99%) were used without processing. The chemicals were checked chromatographically for the content of the main component and the results were consistent (or better) than those declared by the supplier. Solutions were prepared by weight using an analytical balance. The accuracy of the concentration of mixtures is confident as being ≥0.0001 mol fraction.

Speeds of sound were determined using a computer-operated OPBOX 2.1 (Optel, Wrocław, Poland) apparatus applying the time of flight method, working with absolute accuracy better then ±0.2 m·s^−1^ and precision of similar order. Measurements are based on the determination of the the time that the acoustic signal with a frequency of 8 MHz takes to pass through a sample of known length. Temperature during measurements was stabilized by a precision Julabo F25-ME (Germany) thermostat with an accuracy of ±0.01 °C, while the uncertainty of temperature was about 0.05 °C, checked using a precise mercury thermometer.

The density was measured using an Automatic Density Meter DDM 2911 (Rudolph Research Analytical, Hackettstown, USA) with an accuracy ca. ±0.05 kg·m^−3^, equipped with the built-in thermostat which guarantees the temperature stabilization of ±0.01 °C and temperature accuracy of about 0.05 °C.

**Fig. 1:**
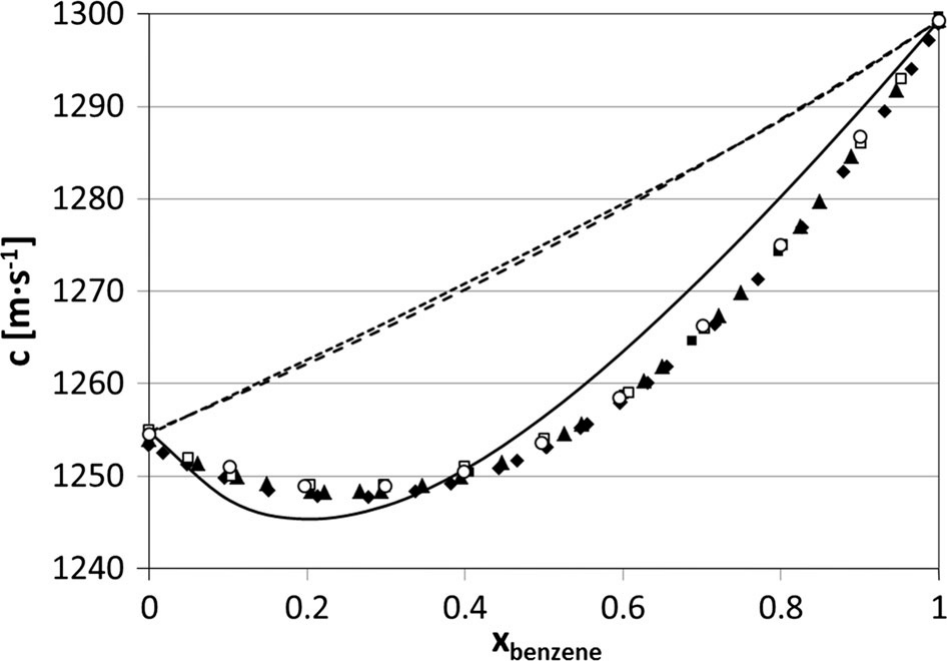
Speeds of sound versus composition in cyclohexane + benzene solutions at 25 °C. *Empty circles*, experimental data; *solid line*, this work; *broken line*, two-component Natta–Baccaredda model (Eq. 8), *dotted line*, ideal values of the speeds of sound (Eq. 11). *Filled triangles*, the data from [11]; *empty squares*, the data from [17]; *filled squares*, the data from [53]; *filled diamonds*, the data from [54]. The average absolute deviations of the models from experimental data are 4.5 m·s^−1^ (ideal speeds of sound from Eq. 11), 4.3 m·s^−1^ (Natta–Baccaredda model) and 1.2 m·s^−1^ (this model)

**Fig. 2:**
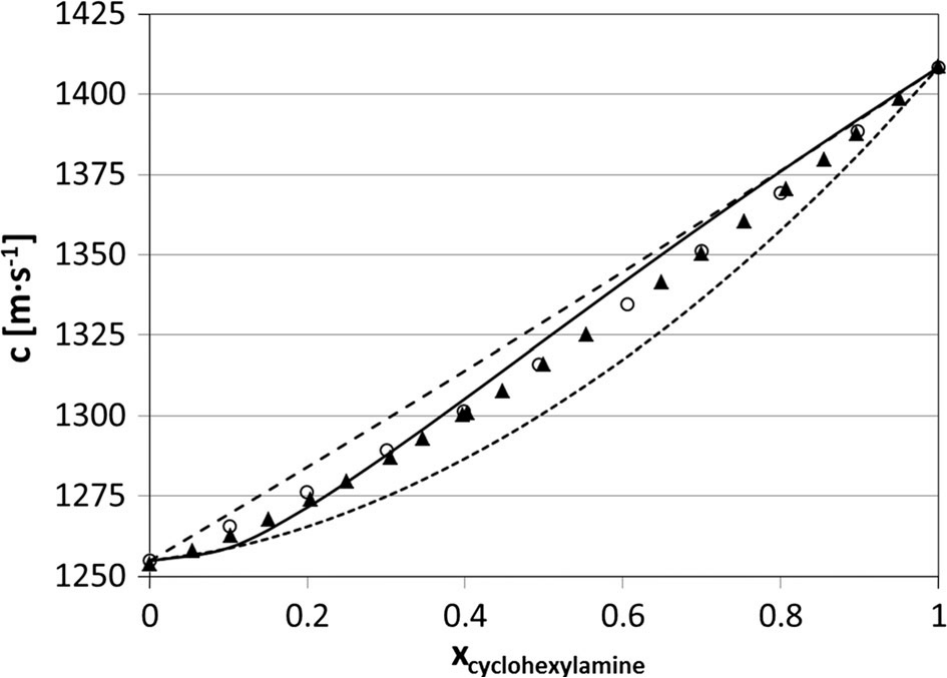
Speeds of sound versus composition in the cyclohexane + cyclohexylamine solutions at 25 °C. For details refer to the caption of Fig. 1. *Filled triangles*, the data from [10]. The average absolute deviations of the models from experimental data are 4.1 m·s^−1^ (ideal speeds of sound from Eq. 11), 3.3 m·s^−1^ (Natta–Baccaredda model) and 2.1 m·s^−1^ (this model)

Our results are in very good agreement with those already published [10–54] as illustrated in Table 1 and Figs. 1, 2. However, no data have been found for concentration dependences of sound speeds and densities of the cyclohexane + aniline system at 35 °C.

**Table 1:** Comparison of experimental speed of sounds (*c*) and densities (*ρ*) of pure liquids with literature values at 15, 20, 25, 30 and 35 °C

Liquid	*T* (°C)	*c*/m·s^−1^		*ρ*/kg·m^−3^	
		Exp.	Lit.	Exp.	Lit.
Benzene	15	1344.3	–	884.13	–
	20	1322.7	1322.7 [10, 11]	878.77	878.95 [10, 11]
	25	1299.3	1299.3 [10, 11]	873.49	873.61 [10, 11]
			1298 [12, 13, 16, 18]		873.63 [12, 18]
			1300 [14, 20]		873.56 [13]
			1302.6 [15]		873.58[14]
			1299 [17]		876.5 [15]
			1298.3 [21]		873.64 [16, 22]
					873.6 [17]
					873.75 [20]
					873.5 [21]
	30	1276.0	1276.0 [10, 11]	868.06	868.25 [10, 11]
			1275.5 [19, 22]		868.39 [19, 22]
	35	1252.8	1252.8 [10, 11]	862.59	862.88 [10, 11]
			1259 [12]		862.99 [12]
			1260 [14]		862.96 [14]
Cyclohexane	15	1304.4	–	783.23	–
	20	1278.9	1278.6 [10, 11]	778.56	778.55 [10, 11]
			1278.0 [28]		778.4 [25, 38]
			1279 [34, 38]		778.706 [28]
					778.8 [31]
					778.6 [35]
	25	1254.8	1253.9 [10, 11]	773.89	773.84 [10, 11]
			1255 [12, 13, 16–18, 32, 36]		773.93 [12]
			1254 [14, 27, 29]		773.86 [13, 14, 27, 29]
			1254.6 [15]		777.3 [15]
			1254.4 [21]		773.94 [16, 18, 24, 32, 36]
			1253.71 [23]		773.9 [17, 31, 35]
			1253.6 [24, 28]		773.7 [21]
			1254.95 [26]		773.98 [22]
			1256 [38]		773.87 [23]
					773.82 [26]
					774.018 [28]
					773.8 [37, 38]
	30	1230.9	1229.3 [10, 11]	769.13	769.11 [10, 11]
			1231 [19, 22]		768.98 [19]
			1229.1 [24]		769.14 [22]
			1229.9 [28]		769.21 [24]
			1229 [29]		769.0 [25, 38]
			1228 [30, 33]		769.297 [28]
			1219 [38]		769.12 [29]
					768.3 [30, 33]
					769.3 [31]
					769.2 [35]
	35	1208.0	1205.1 [10, 11]	764.32	764.35 [10, 11, 29]
			1212 [12, 14]		764.68 [12]
			1205.1 [24]		764.46 [14, 27]
			1212 [27]		764.45 [24]
			1205 [29]		764.4 [30, 33]
			1208 [30, 33]		764.5 [31]
					764.38 [37]
Cyclohexylamine	15	1451.5	–	871.35	–
	20	1429.8	1430.5 [10]	866.93	867.39 [10]
	25	1408.4	1408.8 [10]	862.36	862.85 [10]
			1416.4 [40–42]		862.8 [40]
					866.8 [41, 42]
	30	1386.8	1387.3 [10]	857.79	858.31 [10]
			1397.8 [39]		857.71 [39]
	35	1365.1	1366.0 [10]	853.23	853.76 [10]
			1379.2 [39]		853.14 [39]
Aniline	35	1602.4	1595 [43]	1008.59	1008.91 [43]
			1598.3 [44]		1008.4 [44]
			1602.95 [46, 47]		1008.7 [45]
			1604.5 [49]		1008.67 [46, 47]
			1565 [50]		1007.8 [50]
			1594 [51, 52]		1008.8 [51, 52]

## Results and Discussion

The experimental sound speeds, densities, and the quantities calculated using them are collected as the deposited supplementary material.

**Fig. 3:**
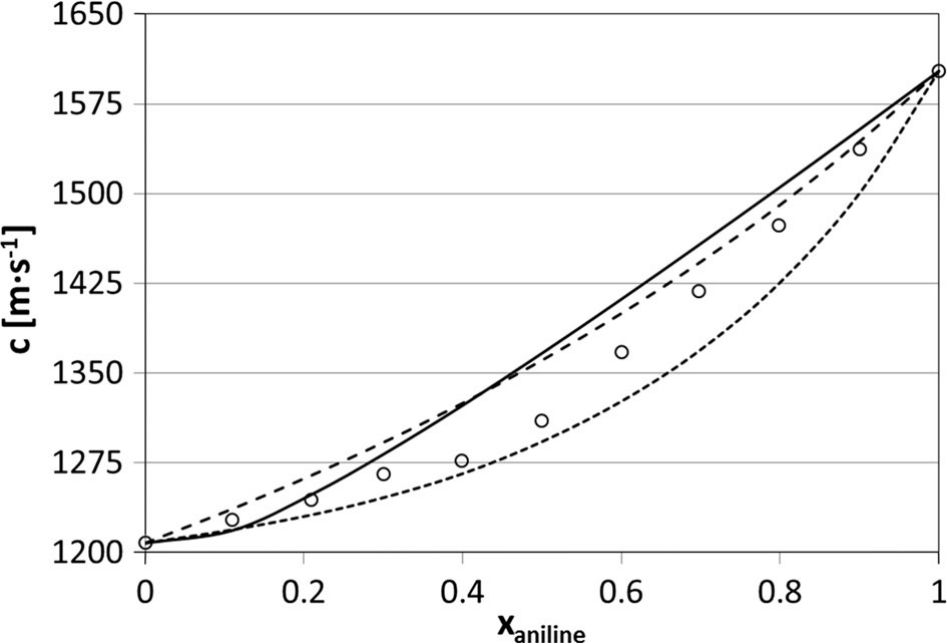
Speeds of sound versus composition in cyclohexane + aniline solutions at 35 °C. For details refer to the caption of Fig. 1. The average absolute deviations of the models from experimental data are 19.9 m·s^−1^ (ideal speeds of sound from Eq. 11), 4.8 m·s^−1^ (Natta–Baccaredda model) and 16.8 m·s^−1^ (this model)

The values of the equilibrium constants of dimerization *K*_dim_ obtained by us are listed in Table 2 and illustrated in Figs. 1–3, for 25 °C (35 °C for aniline + cyclohexane, because the latter system is two-phase below this temperature with the upper critical point located at *x*_c_ = 0.4551 and *T*_c_ = 29.63 °C [55]). In contrast to the alcohol–hydrocarbon systems reported in [2], deviations of the fitted values from the experimental ones are similar over the whole range of concentrations. The results were then used to calculate the basic thermodynamic parameters of the dimerization process according to the well-known relations:

5 $$ \Delta G^{\circ}= - RT\ln K_{\dim } $$

6 $$ \frac{{d\ln K_{\dim } }}{dT} = \frac{{\Delta H}^{\circ}}{{RT^{2} }} $$

7 $$ \Delta S^{\circ} = \frac{{\left( {\Delta H^{\circ} - \Delta G^{\circ}} \right)}}{T} $$

Note, however, that the temperature dependence of *K*_dim_, $\frac{{d\ln K_{\dim } }}{dT}$, is determined with very low accuracy, and therefore both ∆*H*° and ∆*S*° are very imprecise (in particular the former quantity).

**Table 2:** Results of calculations

Solute	Temperature/ °C	*K*_dim_^a^	$\frac{{d\ln K_{\dim } }}{dT}$	∆*H*°/ kJ·mol^−1^	∆*G*°/ kJ·mol^−1^	∆*S*°/ J·mol^−1^·K^−1^
Benzene (∆*c* = 500 m·s^−1^)^*/^	15	0.032	–0.0056	–3.83	8.26	–42.0
	20	0.032		–3.96	8.35	–42.0
	25	0.032		–4.10	8.54	–42.4
	30	0.030		–4.24	8.86	–43.3
	35	0.029		–4.38	9.08	–43.7
Cyclohexylamine (∆*c* = 500 m·s^−1^)	15	0.081	0.0046	3.17	6.44	–11.3
	20	0.077		3.28	7.37	–13.9
	25	0.073		3.40	7.11	–12.4
	30	0.076		3.51	8.15	–15.3
	35	0.080		3.63	6.32	–8.74
Aniline (∆*c* = 1000 m·s^−1^)	35	0.230	–	–	3.70	–12.2

^*/^Values of ∆*c* are fitted with only 100 m·s^−1^ accuracy (see [2]). The approximate relative uncertainties of *K*_dim_, ∆*H*°, ∆*G*° and ∆*S*° are of the order of 10–20%

^a^ This quantity is dimensionless because it was calculated using concentrations expressed in mole fractions in Eq. 2

Independent of the accuracy of the above results, it is clear that ∆*G*° is positive and dimerization is entropically unfavorable (negative ∆*S*°). Dimerization of aniline is much more favored than for benzene or cyclohexylamine (*K*_dim_ much higher, ∆*G* lower than for the latter compounds). Note also that for aniline the difference in sound speeds in the hypothetical pure monomeric and dimeric phases is twice as large, also suggesting much smaller amounts of compressible associated species, i.e. stronger solute–solute interaction.

When analyzing Figs. 1–3, we conclude that there are two regions of concentration where the model yields greater deviations from experimental data: downward in the region of low solute concentrations and upwards at relatively high concentrations. This is seen in particular for benzene solutions (Fig. 1). It is easy to explain this phenomenon: for low concentrations of the polar solute the solute–solvent interactions play an important role, while for high concentrations the competing process can be the formation of associates bigger than dimers.

Deeper analysis leads to the following conclusion. The solute–solvent interactions observed as deviations in dilute systems end at ca. 0.4 mol fraction for benzene, larger than for amines (*x* about 0.2 for aniline and 0.3 for cyclohexylamine). These concentrations correspond to the intersection concentration of experimental and calculated curves. This is consistent with the strengths of solute–solute bonding, proportional to *K*_dim_, which increase in the order benzene < cyclohexylamine < aniline. This is also consistent with expectations: benzene–benzene interactions are relatively weak, the most relevant configuration for dimers in pure liquid C_6_H_6_ and in a mixture with cyclohexane has the T-shape. On the other hand, benzene–cyclohexane interactions are of similar interaction energy [56]. For cyclohexylamine and aniline the solute–solute interactions are mainly electrostatic in nature, which are much stronger. Moreover, amines have a tendency to form associates larger than dimers [57], which is probably responsible for the observed greater difference in sound speeds in the hypothetical phases (Δ*c*) and higher *K*_dim_ values mentioned before.

In Figs. 1–3 two other models are also presented for comparison. Broken lines are for the “classical” Natta–Baccaredda model, where the sound speed of a mixture is calculated from the sound speeds of pure liquids only [4], according to the following relation:

8 $$ c_{\text{calc}} = \frac{{c_{\text{solute}} \cdot c_{\text{solvent}} }}{{\phi_{\text{solute}} \cdot c_{\text{solvent}} + \phi_{\text{solvent}} \cdot c_{\text{solute}} }} $$

This equation has practical use because of its simplicity, but its theoretical basis is weak. The thermodynamically exact equation for ideal compressibility of a solution, recommended by Benson and Kiyohara [58] (after Van Dael [59]) and by Douheret et al. [60], is as follows:

9 $$ \kappa_{S,id} = \sum {\phi_{i} } \left\{ {\kappa_{S,i}^{0} + TV_{i}^{0} \frac{{\left( {\alpha_{i}^{0} } \right)^{2} }}{{C_{p,i}^{0} }}} \right\} - T\left( {\sum {x_{i} V_{i}^{0} } } \right)\left( {\frac{{\sum {\phi_{i} \left( {\alpha_{i}^{0} } \right)^{2} } }}{{\sum {x_{i} C_{p,i}^{0} } }}} \right) $$

where $V_{i}^{0}$, $\alpha_{i}^{0}$ and $C_{p,i}^{0}$ are the molar volume, isobaric thermal expansion coefficient and molar isobaric heat capacity of the pure *i*th component, respectively; *ϕ*_*i*_ is the volume fraction of the *i*th component in the mixture. The values of $\alpha_{i}^{0}$ and $V_{i}^{0}$ were calculated from experimental density data, while those for $C_{p,i}^{0}$ were taken from the literature [61–64].

Combining ideal compressibilities obtained from Eq. 9 and ideal densities calculated as:

10 $$ \rho_{\text{id}} = \frac{{\sum {x_{i} M_{i} } }}{{\sum {x_{i} V_{i}^{0} } }} , $$

where *M*_*i*_ is the molar mass of *i* ^th^ component in the mixture, one can easily calculate the ideal sound speeds in solutions, shown as dotted lines in Figs. 1–3, from:

11 $$ c_{\text{id}} = \sqrt {\frac{1}{{\rho_{\text{id}} \kappa_{{S,{\text{id}}}} }}} $$

There are many models that could be also used to estimate the sound speeds, for example the so-called “molecular sound velocity” or “molecular compressibility” models, as suggested by Nomoto [65]. However, these relations are mainly empirical and have no firm theoretical basis, so we will not apply them in our interpretation.

The model presented here gives similar or better agreement with experimental speeds of sound compared to those predicted by the Natta–Baccaredda or ideal model calculated from Eq. 11, especially for cyclohexane + benzene—see the average deviations listed in the figure’s captions. Interestingly, for the latter system the ideal speeds of sound calculated from Eq. 11 give results rather far from the experimental values, although the two components have both densities and sound speeds in pure components close to each other ($\rho _{{{\text{benzene}}}}^{{25\,^{\,\circ} {\text{C}}}}\,=\,873.5\,{\text{kg}}{\cdot}{\text{m}}^{{ - 3}}$, $\rho _{{{\text{cyclohexane}}}}^{{25\,^{{\text{o}}} {\text{C}}}}\,=\,773.9\,{\text{kg}}{\cdot}{\text{m}}^{{ - 3}}$, $c_{{{\text{benzene}}}}^{{25\,^{{\,\text{o}}} {\text{C}}}}\,=\,1299.3\,{\text{m}}{\cdot}{\text{s}}^{{ - 1}}$, and $c_{{{\text{cyclohexane}}}}^{{25\,^{\,\circ} {\text{C}}}}\,=\,1254.8\,{\text{m}}{\cdot}{\text{s}}^{{ - 1}}$).

One might be surprised that very simple Natta–Baccaredda equation, which includes no adjustable parameters, yields results of similar quality as the much more complex model, which operates with a few fitted parameters, the same situation concerns Eq. 11. It seems that Eq. 11 assumes ideality of the system and measures the deviation of the system from it, while the current calculations assume, inversely, non-ideality of the solution caused by dimerization. Of course, this assumption is rather rough and this explains the inaccuracy of the fittings. Apparently, this inaccuracy is of similar order as that caused by non-ideality as measured in terms of Eq. 11.

The cyclohexane + aniline system has been rarely reported in the literature. From vibrational spectra Chowdhury et al. [66] concluded that aniline forms very weak sandwich-like associates with cyclohexane. It was found that the –NH_2_ group is able to interact with the aromatic π-electrons of the ring. However, electrostatic repulsion prevents attaching another aniline molecule to such a dimer. This is in agreement with our observation that the relatively small deviations of our model from the experiment values occurs at low aniline concentrations.

Also, cyclohexylamine in the cyclohexane system has not often been investigated. Malek et al. [10] suggested that destruction of H-bondings between amine molecules is responsible for deviations of the observed quantities from those predicted by the Prigogine–Flory–Patterson theory.

The system cyclohexane + benzene has been investigated relatively often [67], but the interpretations are not unanimous. The heats of mixing for C_6_H_12_ + C_6_H_6_ and the vapor pressure of these solutions show that this system is far from regular [68], although the calculations of Milano et al. imply only slight non-ideal behavior [56]. Non-ideality of this system was also stressed by Bottomley and Coopes based on the vapor pressure method [69]. Recently, Ninkovic et al. [70] found from crystal structures and ab initio calculations that the cyclohexane–benzene interaction energy is significantly stronger than that for the benzene dimer, indicating the importance of aliphatic–aromatic interactions. This is probably responsible for the relatively large deviations of the Natta–Baccaredda and Benson–Kiyohara models from experiment results observed over the whole range of concentrations for benzene solutions. According to Domingues et al. [51], cyclohexane alters the local order in liquid benzene, as suggested by Narten [71] and further discussed by Kiyohara et al. [54]. They suppose that cyclohexane, which exists both in chair and boat forms, acts like a structure breaker, leading to more compressible structures in solution. From self-diffusion measurements it was only concluded that there are aggregates of molecules present in these solutions [72]. On the other hand, Ward interpreted the X-ray diffraction results in terms of formation of an emulsion-type solution, in which the disperse phase is too small to manifest a Tyndall effect [73].

## Conclusions

Although operating with very simple and rough assumptions, the model applied in this work seems to yield rational values of the dimerization constants of the solutes in cyclohexane. It was found that the weakest interactions are solute–solute interactions in benzene and the strongest are in aniline solutions. For all of the systems, formation of dimers is not favored thermodynamically (positive ∆*G*° and negative ∆*S*°). The effect of solute–solvent association, observed in low concentrations, is relatively strong for benzene and weaker for the other solutes. The same situation concerns the solute–solute interactions observed at high benzene concentrations, where additional stacking association effects can be assumed, similar to the systems containing cyclohexylamine and aniline, where strong solute–solute interactions result in formation of trimers, tetramers, etc., as well as cyclic associates.

## Electronic supplementary material

Below is the link to the electronic supplementary material.

Supplementary material 1 (DOC 541 kb)
