# Supplementary material for: Dimerization Constants from Acoustic Measurements: Solutions of Benzene, Cyclohexylamine and Aniline in Cyclohexane
Source: J Solution Chem. 2017 Jul 18;46(7):1501–13. doi: 10.1007/s10953-017-0656-1 (PMC5527082; doi:10.1007/s10953-017-0656-1)
Supplement: Supplementary file 1 — Supplementary material 1 (DOC 541 kb) [file 10953_2017_656_MOESM1_ESM.doc]

**Supplementary material for:**

**Dimerization Constants from Acoustic Measurements: Solutions of Benzene, Cyclohexylamine and Aniline in Cyclohexane**

**Andrzej Burakowski · Jacek Gliński**

Faculty of Chemistry, University of Wrocław

F. Joliot-Curie 14, 50-383 Wrocław

**Table S1** Speeds of sound, *c* / m·s^–1^, for benzene + cyclohexane mixtures at 15–35 °C

| *X*_benzene_ | Speed of sound, *c* / m·s^–1^ | | | | |
| --- | --- | --- | --- | --- | --- |
|  | 15 °C | 20 °C | 25 °C | 30 °C | 35 °C |
| 0.0000 | 1304.4 | 1278.9 | 1254.8 | 1230.9 | 1208.0 |
| 0.1020 | 1297.8 | 1274.6 | 1251.0 | 1228.0 | 1204.9 |
| 0.1972 | 1295.2 | 1272.4 | 1248.9 | 1225.8 | 1202.9 |
| 0.2991 | 1294.9 | 1272.0 | 1248.9 | 1225.8 | 1202.9 |
| 0.3985 | 1295.7 | 1273.2 | 1250.5 | 1227.9 | 1205.4 |
| 0.4975 | 1298.8 | 1276.4 | 1253.6 | 1231.1 | 1208.7 |
| 0.5954 | 1303.3 | 1281.2 | 1258.5 | 1236.2 | 1213.7 |
| 0.7011 | 1310.9 | 1288.8 | 1266.3 | 1243.8 | 1221.4 |
| 0.7996 | 1319.7 | 1297.6 | 1275.0 | 1253.0 | 1230.8 |
| 0.9002 | 1331.5 | 1309.2 | 1286.7 | 1264.2 | 1241.8 |
| 1.0000 | 1344.3 | 1322.7 | 1299.3 | 1276.0 | 1252.8 |

Standard uncertainties *u* are *u*(*x*) = 0.00005, *u*(*T*) = 0.05 °C, *u*(*c*) = 0.2 m⋅s^−1^

**Table S2** Densities, *ρ* / kg·m^–3^, for benzene + cyclohexane mixtures at 15–35 °C

| *X*_benzene_ | Density, *ρ* / kg·m^–3^ | | | | |
| --- | --- | --- | --- | --- | --- |
|  | 15 °C | 20 °C | 25 °C | 30 °C | 35 °C |
| 0.0000 | 783.23 | 778.56 | 773.89 | 769.13 | 764.32 |
| 0.1020 | 789.88 | 785.12 | 780.45 | 775.59 | 770.69 |
| 0.1972 | 796.67 | 791.87 | 787.15 | 782.26 | 777.32 |
| 0.2991 | 804.70 | 799.87 | 795.10 | 790.16 | 785.18 |
| 0.3985 | 813.14 | 808.15 | 803.43 | 798.45 | 793.42 |
| 0.4975 | 822.45 | 817.52 | 812.62 | 807.62 | 802.54 |
| 0.5954 | 832.45 | 827.48 | 822.50 | 817.42 | 812.27 |
| 0.7011 | 844.57 | 839.52 | 834.47 | 829.30 | 824.10 |
| 0.7996 | 856.64 | 851.53 | 846.43 | 841.15 | 835.86 |
| 0.9002 | 869.92 | 864.72 | 859.51 | 854.16 | 848.78 |
| 1.0000 | 884.13 | 878.77 | 873.49 | 868.06 | 862.59 |

Standard uncertainties *u* are *u*(*x*) = 0.00005, *u*(*T*) = 0.05 °C, *u*(*d*) = 0.05 kg⋅m^−3^

**Table S3** Adiabatic compressibility coefficients, *κ_S_* × 10^10^ / Pa^–1^, for benzene + cyclohexane mixtures at 15–35 °C

| *X*_benzene_ | Adiabatic compressibility coefficient, *κ_S_* × 10^10^ / Pa^–1^ | | | | |
| --- | --- | --- | --- | --- | --- |
|  | 15 °C | 20 °C | 25 °C | 30 °C | 35 °C |
| 0.0000 | 7.504 | 7.854 | 8.206 | 8.581 | 8.965 |
| 0.1020 | 7.517 | 7.840 | 8.188 | 8.550 | 8.937 |
| 0.1972 | 7.482 | 7.801 | 8.144 | 8.508 | 8.891 |
| 0.2991 | 7.411 | 7.727 | 8.063 | 8.423 | 8.802 |
| 0.3985 | 7.325 | 7.634 | 7.960 | 8.307 | 8.675 |
| 0.4975 | 7.208 | 7.508 | 7.830 | 8.170 | 8.529 |
| 0.5954 | 7.072 | 7.363 | 7.676 | 8.005 | 8.358 |
| 0.7011 | 6.890 | 7.172 | 7.473 | 7.795 | 8.134 |
| 0.7996 | 6.703 | 6.974 | 7.267 | 7.572 | 7.897 |
| 0.9002 | 6.484 | 6.747 | 7.028 | 7.326 | 7.640 |
| 1.0000 | 6.259 | 6.504 | 6.782 | 7.075 | 7.386 |

Standard uncertainties *u* are *u*(*x*) = 0.00005, *u*(*T*) = 0.1 °C, *u*(*κ_S_*) = 2 × 10^−13^ Pa^−1^

**In all of the following figures all the lines are drawn arbitrarily, and are only to guide the eye.**


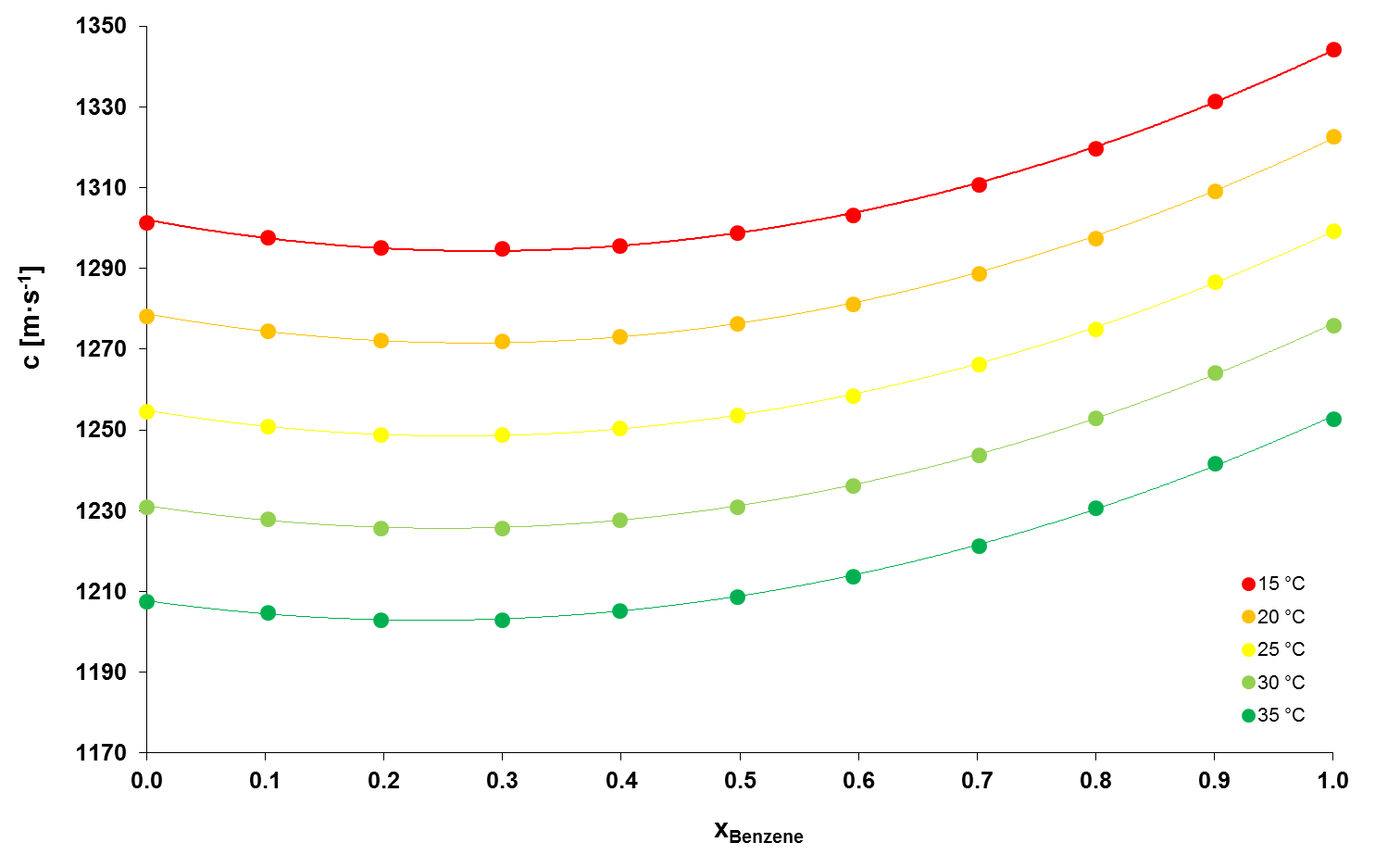


**Fig. S1** Speeds of sound, *c* / m·s^–1^, for benzene + cyclohexane mixtures at 15–35 °C


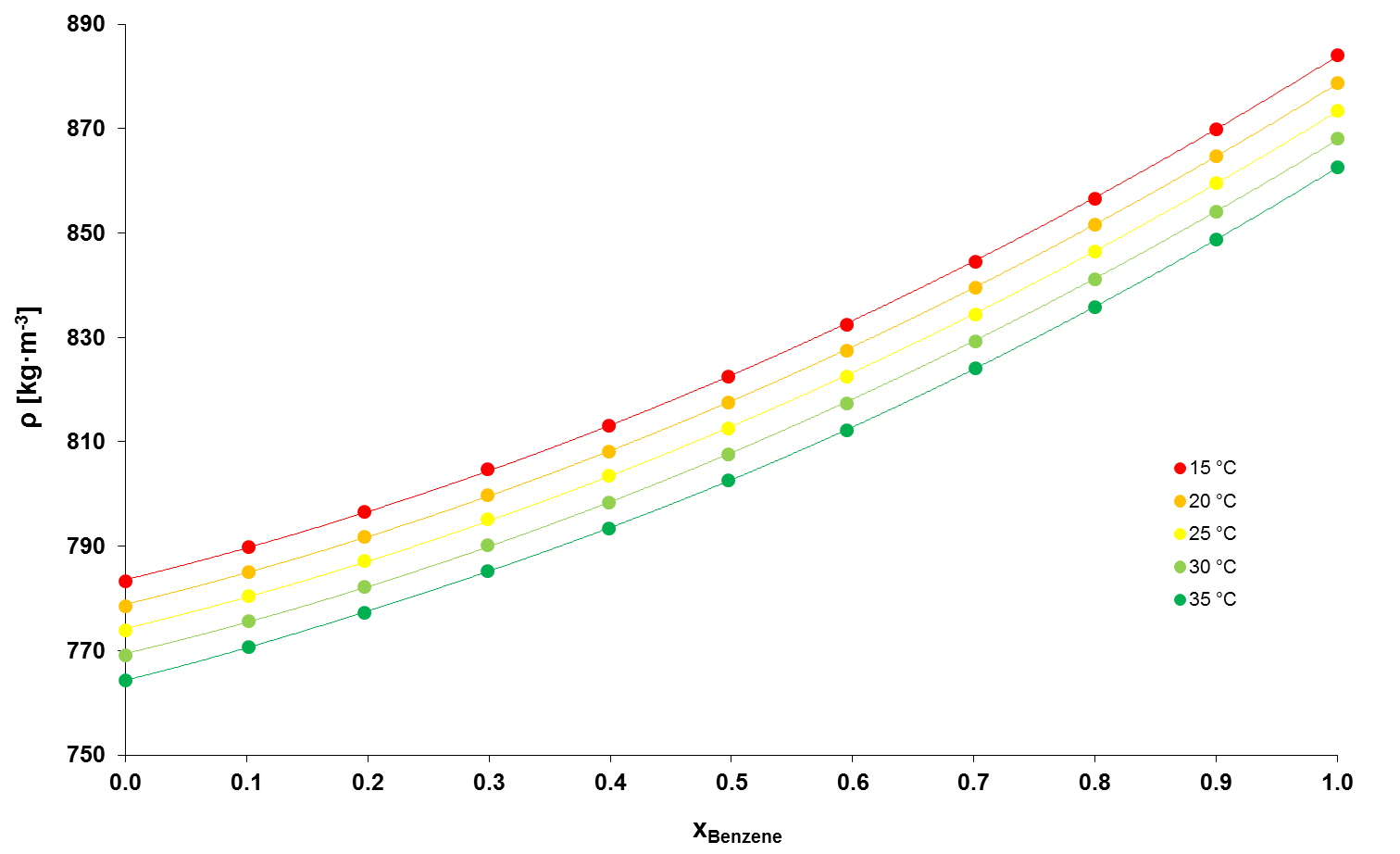


**Fig. S2** Densities, *ρ* / kg·m^–3^, for benzene + cyclohexane mixtures at 15–35 °C


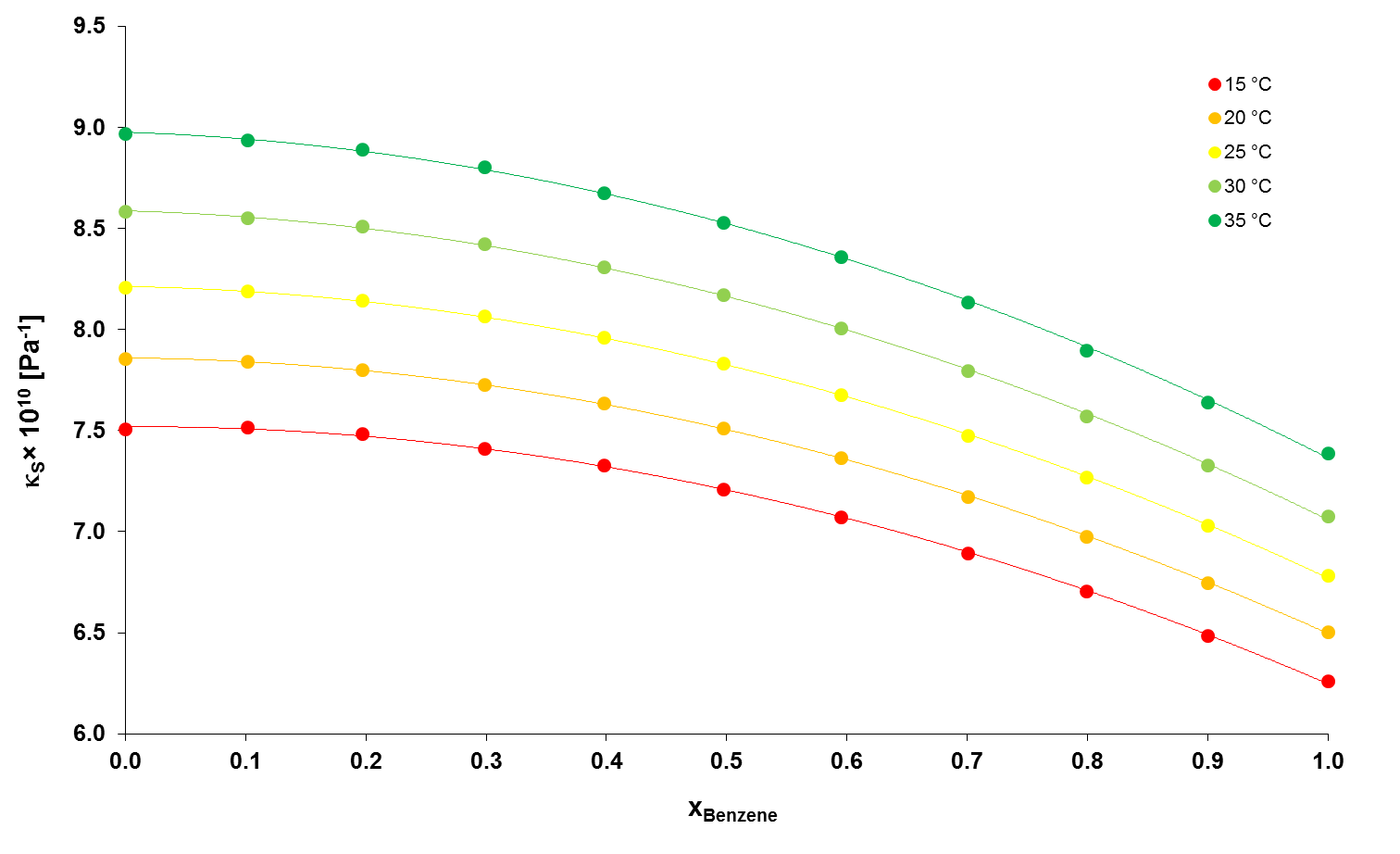


**Fig. S3** Adiabatic compressibility coefficients, *κ_S_* × 10^10^ / Pa^–1^, for benzene + cyclohexane mixtures at 15–35 °C

**Table S4** Speeds of sound, *c* / m·s^–1^, for cyclohexylamine + cyclohexane mixtures
at 15–35 °C

| *X*_cyclohexylamine_ | Speed of sound, *c* / m·s^–1^ | | | | |
| --- | --- | --- | --- | --- | --- |
|  | 15 °C | 20 °C | 25 °C | 30 °C | 35 °C |
| 0.0000 | 1304.4 | 1278.9 | 1254.8 | 1230.9 | 1208.0 |
| 0.1010 | 1311.7 | 1287.3 | 1265.5 | 1240.6 | 1217.4 |
| 0.1989 | 1322.7 | 1297.6 | 1276.4 | 1253.1 | 1230.0 |
| 0.3007 | 1335.0 | 1312.0 | 1289.3 | 1266.3 | 1243.4 |
| 0.3984 | 1346.1 | 1323.9 | 1301.5 | 1278.5 | 1255.9 |
| 0.4932 | 1360.8 | 1338.3 | 1316.0 | 1293.4 | 1271.1 |
| 0.6059 | 1379.0 | 1358.8 | 1334.6 | 1312.2 | 1290.1 |
| 0.6997 | 1395.0 | 1372.9 | 1351.3 | 1330.1 | 1309.3 |
| 0.7995 | 1413.0 | 1393.6 | 1369.2 | 1347.9 | 1328.0 |
| 0.8980 | 1431.7 | 1409.9 | 1388.5 | 1366.7 | 1345.0 |
| 1.0000 | 1451.5 | 1429.8 | 1408.4 | 1386.8 | 1365.1 |

Standard uncertainties *u* are *u*(*x*) = 0.00005, *u*(*T*) = 0.05 °C, *u*(*c*) = 0.2 m⋅s^−1^

**Table S5** Densities, *ρ* / kg·m^–3^, for cyclohexylamine + cyclohexane mixtures
at 15–35 °C

| *X*_cyclohexylamine_ | Density, *ρ* / kg·m^–3^ | | | | |
| --- | --- | --- | --- | --- | --- |
|  | 15 °C | 20 °C | 25 °C | 30 °C | 35 °C |
| 0.0000 | 783.23 | 778.56 | 773.89 | 769.13 | 764.32 |
| 0.1010 | 791.22 | 786.58 | 781.91 | 777.18 | 772.41 |
| 0.1989 | 799.49 | 794.87 | 790.20 | 785.46 | 780.70 |
| 0.3007 | 808.43 | 803.78 | 799.11 | 794.37 | 789.63 |
| 0.3984 | 817.15 | 812.50 | 807.82 | 803.11 | 798.39 |
| 0.4932 | 825.67 | 821.05 | 816.39 | 811.68 | 806.96 |
| 0.6059 | 835.87 | 831.30 | 826.64 | 821.95 | 817.26 |
| 0.6997 | 844.41 | 839.84 | 835.20 | 830.54 | 825.87 |
| 0.7995 | 853.40 | 848.87 | 844.25 | 839.61 | 834.98 |
| 0.8980 | 862.26 | 857.77 | 853.18 | 848.59 | 844.00 |
| 1.0000 | 871.35 | 866.93 | 862.36 | 857.79 | 853.23 |

Standard uncertainties *u* are *u*(*x*) = 0.00005, *u*(*T*) = 0.05 °C, *u*(*d*) = 0.05 kg⋅m^−3^

**Table S6** Adiabatic compressibility coefficients, *κ_S_* × 10^10^ / Pa^–1^, for cyclohexylamine
 + cyclohexane mixtures at 15–35 °C

| *X*_cyclohexylamine_ | Adiabatic compressibility coefficient, *κ_S_* × 10^10^ / Pa^–1^ | | | | |
| --- | --- | --- | --- | --- | --- |
|  | 15 °C | 20 °C | 25 °C | 30 °C | 35 °C |
| 0.0000 | 7.504 | 7.854 | 8.206 | 8.581 | 8.965 |
| 0.1010 | 7.346 | 7.671 | 7.986 | 8.361 | 8.736 |
| 0.1989 | 7.150 | 7.472 | 7.768 | 8.108 | 8.467 |
| 0.3007 | 6.941 | 7.228 | 7.528 | 7.851 | 8.191 |
| 0.3984 | 6.754 | 7.022 | 7.308 | 7.618 | 7.941 |
| 0.4932 | 6.540 | 6.800 | 7.073 | 7.364 | 7.670 |
| 0.6059 | 6.292 | 6.515 | 6.791 | 7.066 | 7.351 |
| 0.6997 | 6.086 | 6.317 | 6.557 | 6.805 | 7.063 |
| 0.7995 | 5.869 | 6.066 | 6.318 | 6.556 | 6.791 |
| 0.8980 | 5.658 | 5.865 | 6.080 | 6.309 | 6.549 |
| 1.0000 | 5.448 | 5.642 | 5.846 | 6.062 | 6.289 |

Standard uncertainties *u* are *u*(*x*) = 0.00005, *u*(*T*) = 0.1 °C, *u*(*κ_S_*) = 2 × 10^−13^ Pa^−1^


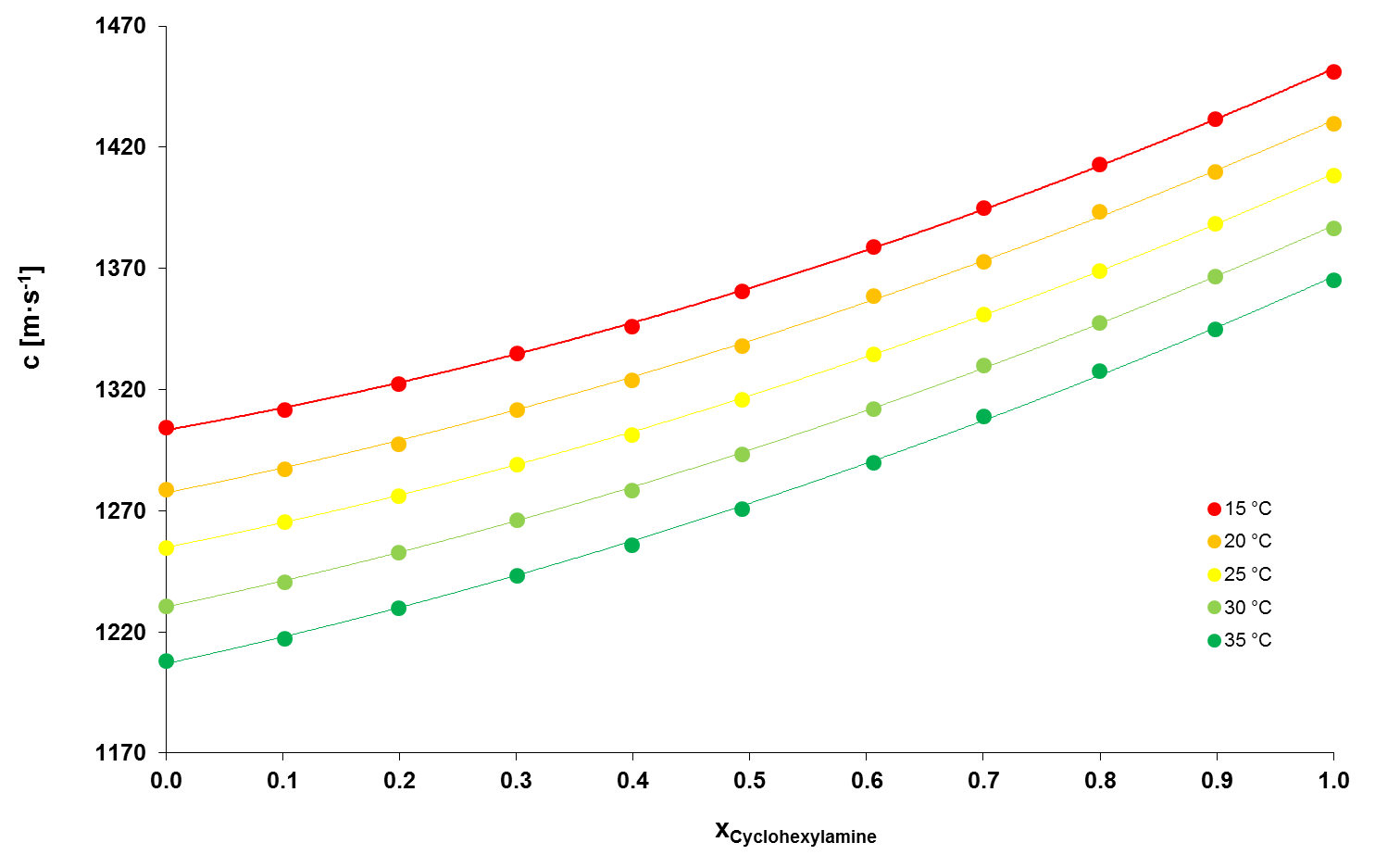


**Fig. S4** Speeds of sound, *c* / m·s^–1^, for cyclohexylamine + cyclohexane mixtures
at 15–35 °C


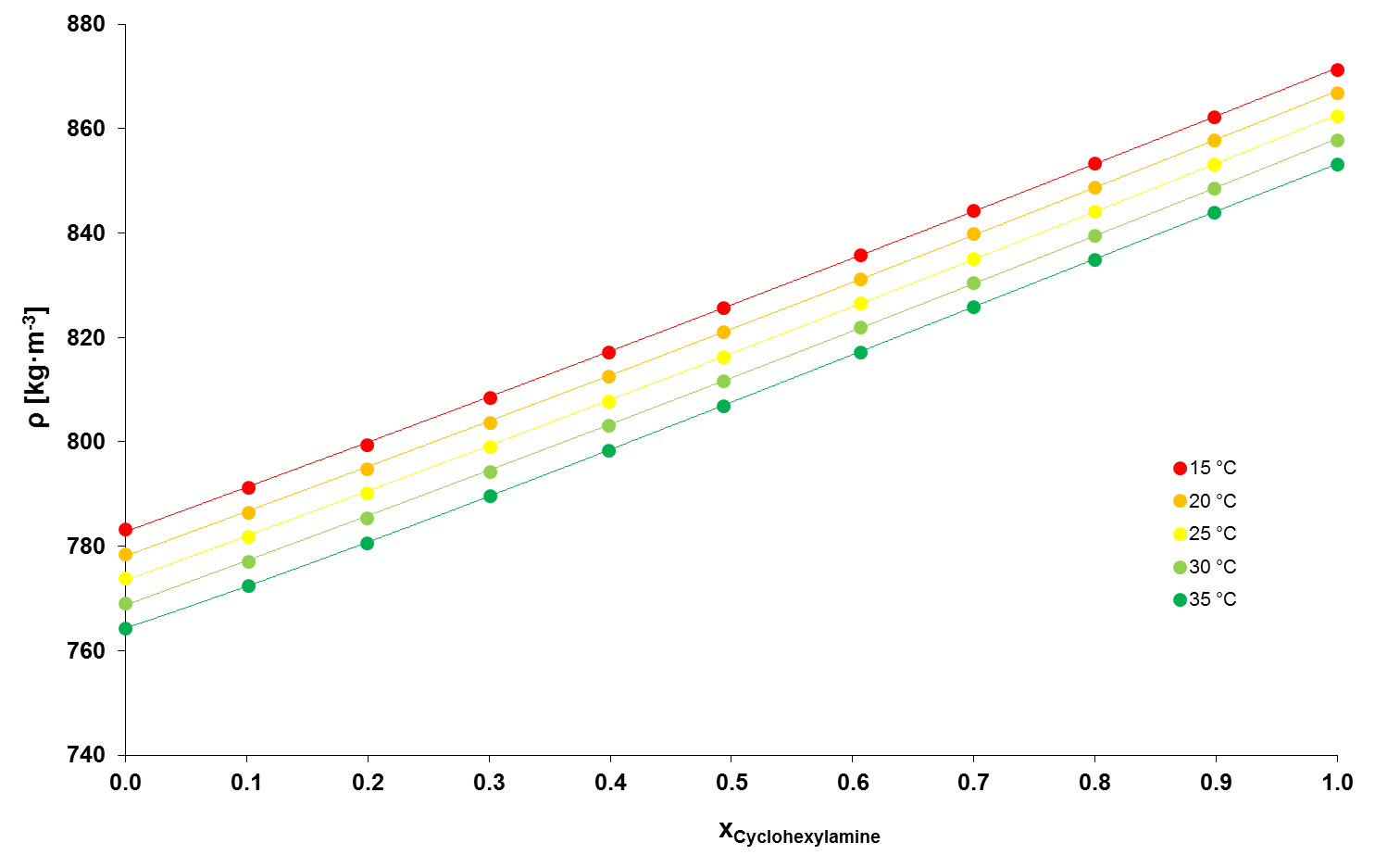


**Fig. S5** Densities, *ρ* / kg·m^–3^, for cyclohexylamine + cyclohexane mixtures
at 15–35 °C


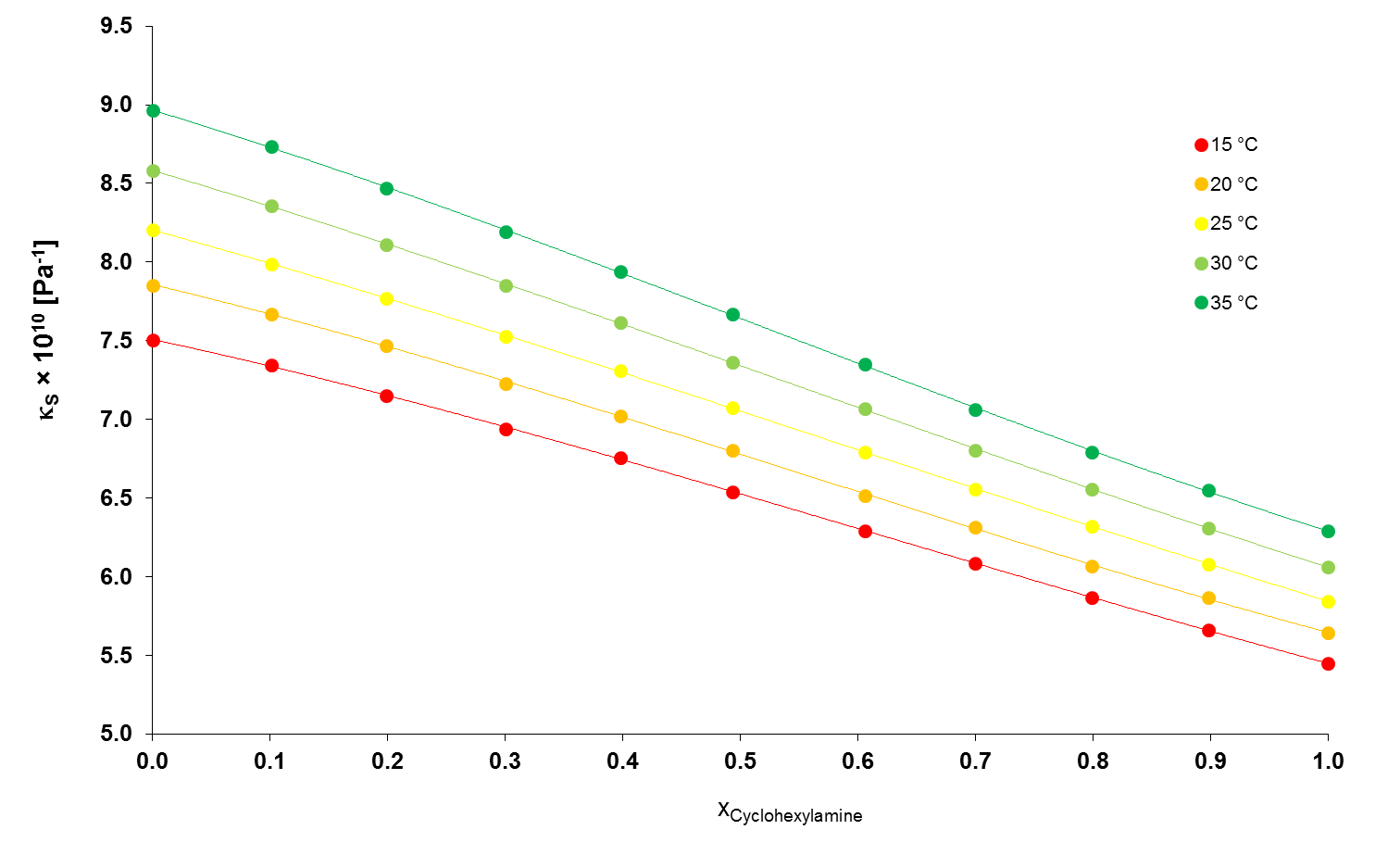


**Fig. S6** Adiabatic compressibility coefficients, *κ_S_* × 10^10^ / Pa^–1^, for cyclohexylamine
 + cyclohexane mixtures at 15–35 °C

**Table S7** Speeds of sound. *c* / m·s^–1^, densities, *ρ* / kg·m^–3^, and adiabatic compressibility coefficients, *κ_S_* × 10^10^ / Pa^–1^, for aniline + cyclohexane mixtures at 35 °C

| *X*_aniline_ | Speed of sound,  *c* / m·s^–1^ | Densities,  *ρ* / kg·m^–3^ | Adiabatic compressibility coefficients, *κ_S_* × 10^10^ / Pa^–1^ |
| --- | --- | --- | --- |
| 0.0000 | 1208.0 | 764.32 | 8.965 |
| 0.1101 | 1227.6 | 784.66 | 8.456 |
| 0.2095 | 1244.3 | 805.48 | 8.018 |
| 0.3003 | 1265.4 | 825.72 | 7.563 |
| 0.3991 | 1276.8 | 848.95 | 7.226 |
| 0.4996 | 1310.2 | 872.54 | 6.676 |
| 0.6002 | 1367.4 | 897.67 | 5.958 |
| 0.6973 | 1418.8 | 922.96 | 5.382 |
| 0.7979 | 1473.7 | 950.32 | 4.845 |
| 0.9006 | 1536.8 | 979.39 | 4.323 |
| 1.0000 | 1602.4 | 1008.59 | 3.861 |

Standard uncertainties *u* are *u*(*x*) = 0.00005, *u*(*T*) = 0.05 °C, *u*(*T_κs_*) = 0.1 °C, *u*(*c*) = 0.2 m⋅s^−1^,
*u*(*d*) = 0.05 kg⋅m^−3^, *u*(*κ_S_*) = 2 × 10^−13^ Pa^−1^


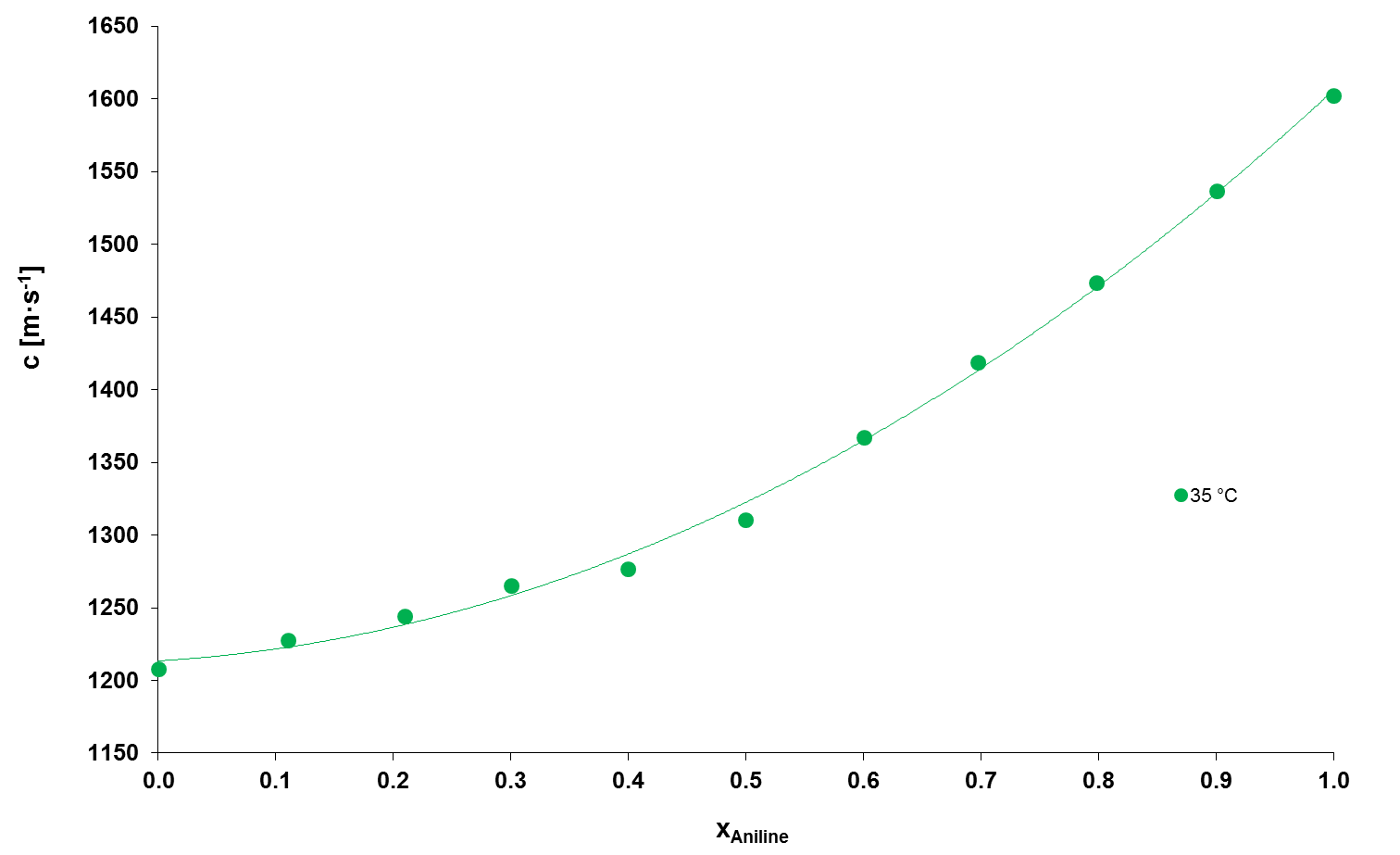


**Fig. S7** Speeds of sound, *c* / m·s^–1^, for aniline + cyclohexane mixtures at 35 °C


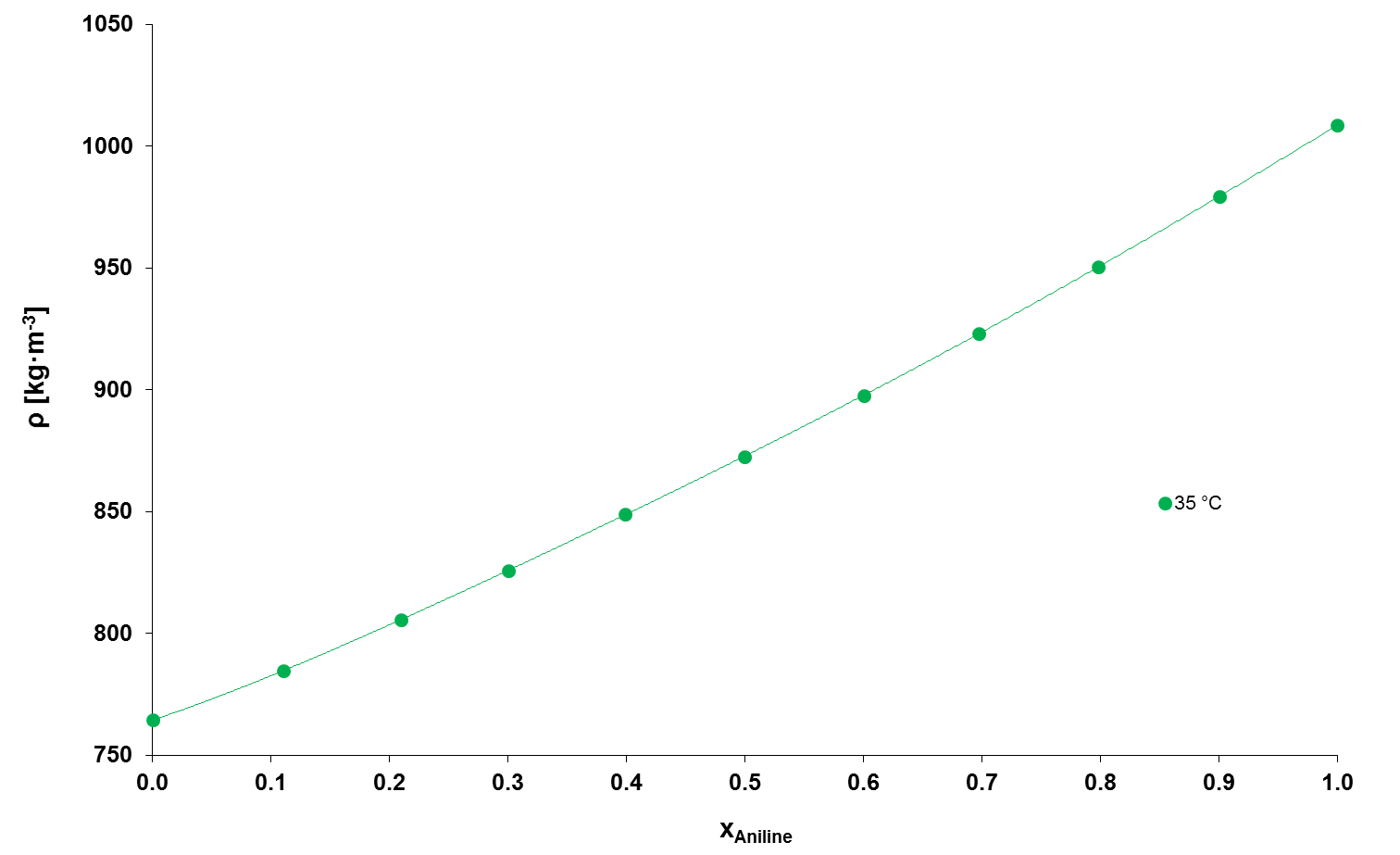


**Fig. S8** Densities, *ρ* / kg·m^–3^, for aniline + cyclohexane mixtures at 35 °C


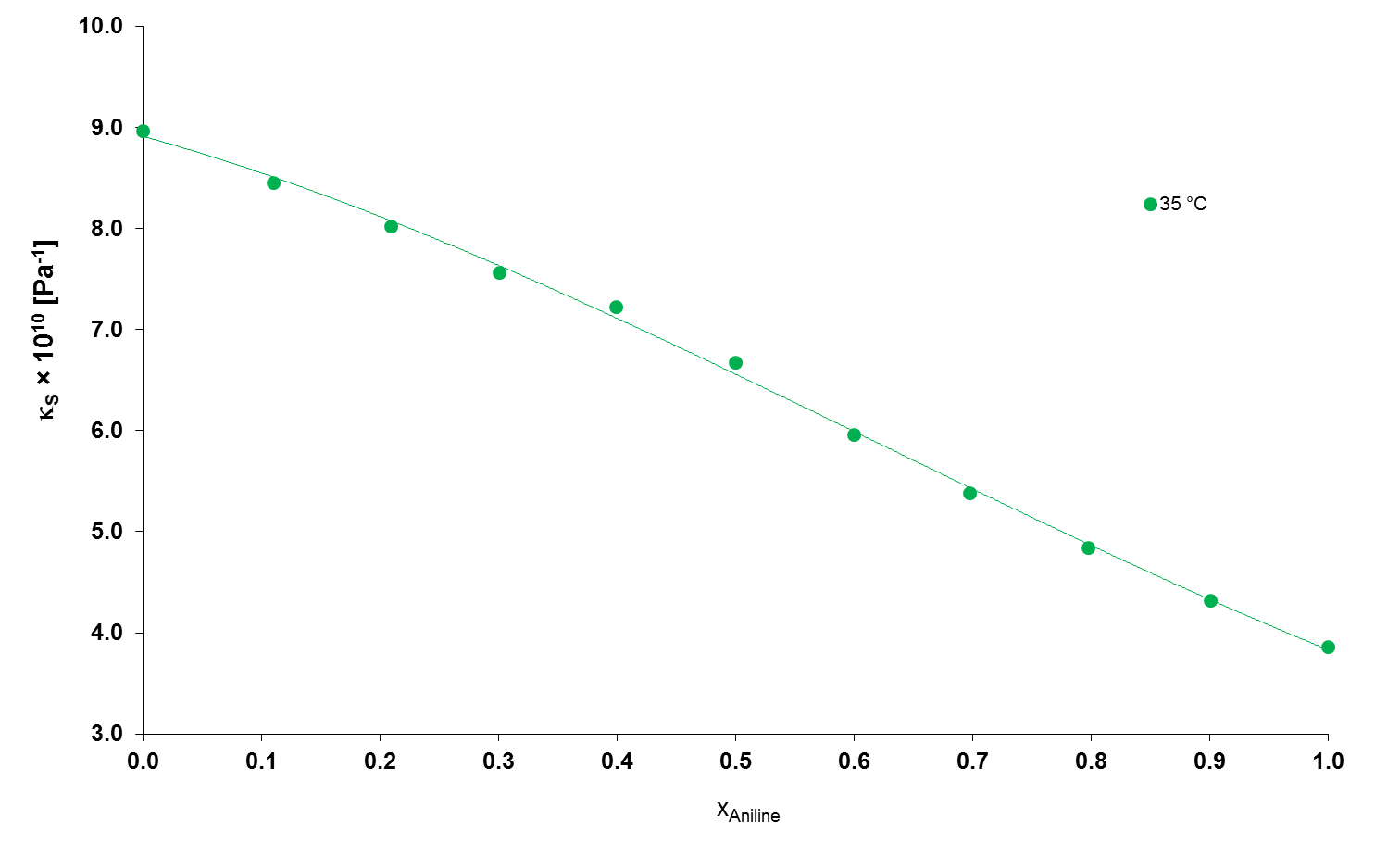


**Fig. S9** Adiabatic compressibility coefficients, *κ_S_* × 10^10^ / Pa^–1^, for aniline + cyclohexane mixtures at 35 °C
